# Effect of diabetic counseling based on conversation map as compared to routine counseling on diabetes management self-efficacy and diabetic distress among patients with diabetes in Pakistan: a randomized controlled trial (study protocol)

**DOI:** 10.1186/s12889-019-7266-3

**Published:** 2019-07-08

**Authors:** Rubina Qasim, Sarfaraz Masih, Mehwish Hussain, Akhter Ali, Ajmal Khan, Yousaf Shah, Hakim Shah, Mohammad Tahir Yousafzai

**Affiliations:** 1 Institute of Nursing, Dow University of Health Sciences, Ojha Campus, Karachi, Pakistan; 2 Nursing Education Services, The Indus Hospital, Karachi, Pakistan; 3 Department of Research, Dow University of Health Sciences, Karachi, Pakistan; 4 National Institute of Diabetes and Endocrinology, Dow University of Health Sciences, Karachi, Pakistan; 5 Department of Pediatrics and Child Health, Aga Khan University, Karachi, Pakistan

**Keywords:** Diabetes, Type 2 diabetes mellitus, Health education, Conversation map, Diabetes management, Diabetic distress

## Abstract

**Background:**

Novel interactive and pictorial health education tool named Diabetes Conversation Map (DCM) might be effective for the improvement of diabetes management self-efficacy (DMSE) and diabetes distress (DD) among diabetic patients in lower middle-income setting. This study protocol will assess the effectiveness of DCM as compared to routine care (RC) to improve DMSE, decrease DD and glycated hemoglobin (HbA1c) among patients with type 2 diabetes (T2DM).

**Methods:**

This will be two arms randomized controlled trial, conducted at national institute of diabetes and endocrinology (NIDE) in Karachi, Pakistan. A sample of 120 T2DM patients of age 30–60 years with suboptimal diabetes control will be screened through eligibility criteria and DD screening tool. Patients who fulfill the eligibility criteria and have diabetes distress will be randomized into interventional and control arm. The intervention arm will receive four education sessions (40 min each) using DCM for 4 weeks duration of enrollment. Controlled arm will receive RC. DMSE and DD will be measured using the validated Likert tools at baseline and after 3 months of enrollment. Latest results of HbA1c will be retrieved from the respective medical record files at baseline and 3 months. Change in DMSE, DD scores and HbA1c levels within groups (pre-post) and between the groups after 3 months of enrollment will be compared. Multivariable linear regression will be conducted to adjust for any potential confounders.

**Discussion:**

In a study in UK, 70% of the patients with diabetes reported DCM had helped them in controlling their diabetes and recommended this method to teach other patients with diabetes also. In China, a study found that patients with diabetes who received DCM based education had significantly lower DD and significantly higher diabetes empowerment score after six months of the intervention as compared to the traditional counselling. A cross sectional study conducted in Pakistan also demonstrated that teaching based on DCM was useful in improving the knowledge, attitude and practices of patients with T2DM. Besides, no other study has evaluated the effectiveness of these novel tools for DMSE and diabetes distress DD in well-designed, sufficiently powered clinical trials.

**Trial registration:**

ClinicalTrials.gov Identifier: NCT03747471. Date of registration: Nov 20. 2018. Version and Date of Protocol: Version 1, IRB Approval date 28 June 2018.

**Electronic supplementary material:**

The online version of this article (10.1186/s12889-019-7266-3) contains supplementary material, which is available to authorized users.

## Background

During the last three decades, the age standardized prevalence of T2DMT2DM has doubled from 4.7% in 1980 to 8.5% in 2014 [1].

The prevalence adjusted for the world by International Diabetes Federation (IDF) is 24% in Saudi Arabia, 23% in Kuwait and Qatar each, 7.8% in Australia, 9.2% in USA, 5% in UK, 9.1% in India, 6.3% in Bangladesh, 9.9% in Iran, 8.3% in Afghanistan, 9% in China, and 7.9% in Pakistan [2].

Uncontrolled diabetes can cause serious acute complications associated with either abnormally high blood glucose resulting in diabetic ketoacidosis (DKA) and hyperosmolar coma or abnormally low blood glucose level resulting in seizures and unconsciousness. Poorly controlled diabetes in the long run causes serious damage to the heart, kidneys, blood vessels, nerves and eyes [3]. According to an estimate, 1.5 million deaths were caused by diabetes in 2012. The percentage of deaths attributable to high blood glucose or diabetes that occurs prior to age 70 is higher in low- and middle-income countries than in high-income countries [2].

As diabetes mellitus affect all aspects of life, its care plan is interlinked with daily actions of the diseased person and hence the diabetic patients also play an important role in the control and management of their disease [4]. DMSE is considered as an important pre-requisite for the success of diabetic control and self-management [5]. Also, it is considered as an independent part of basic skills. Self-Efficacy (SE) is a social cognitive theory proposed by Albert Bandura in 1977. According to Bandura, self-efficacy is a belief in one’s ability to influence events that affects one’s life and control over the way these events are experienced [6]. It further argues that individuals will act when they believe they are able to do it and will avoid any action which they believe they may not be able to perform. Furthermore, the theory proposes that self-efficacy plays an important role in predicting the individuals’ behavior. A study conducted among diabetic patients in Iran, reported strong positive correlation between self-efficacy and diabetes self-care management [7].

On the other hand, the stress associated with diabetes management, its complications or risk of developing complications and living with diabetes itself has the potential of causing significant emotional distress among patients with diabetes. In 1995, the concept of DD was put forward by Polonsky and his team to highlight the negative emotional impact of living with diabetes [8]. Studies from the developed world such as Europe, USA and Australia have shown that about a quarter of the diabetic adult population are experiencing DD [9–11]. Similarly, research among patients with diabetes has shown association between reduction in DD and significant improvement in blood glucose levels [12, 13].

Traditionally, patients with diabetes used to receive education related to their disease process, metabolic control, exercise and diabetic diet through didactic lectures, brochures, pamphlets, and face to face counseling [14]. Among novel methods, Diabetes Conversation Maps (DCM) are considered as useful tools for the educational empowerment of diabetic patients [15]. These are interactive pictorial tools designed in such a way that it does not need formal education for common understanding. In developing countries like Pakistan, where the literacy rate is very less, these tools might prove to be effective for the control of diabetes and its associated complications [16]. DCM have been used and tested in several countries predominantly in the developed world. In the developing region, while it has shown some impact during the observational studies, literature generated from randomized control trials is still limited. Patients in the developing region has different level of education and environmental exposures such as access to internet and social media, they have different socio-demographic characteristics and hence might behave differently from their counterparts in the developed world. Therefore, there is a need of well-designed randomized controlled trial to evaluate the effectiveness of DCM versus routine diabetic care (RC) in DMSE and DD in a low middle income setting of Karachi, Pakistan.

## Methods/design

### Aim of the study

The aim of this study protocol is to assess the effectiveness of DCM as an educational tool in comparison to routine diabetic counseling for improving the DMSE and DD among patients with T2DM.

### Study design and randomization

This will be two parallel arms randomized controlled trial conducted in a public sector tertiary care hospital of Karachi, Pakistan. The randomization will be performed through the generation of random digit numbers from 1 to 120 using Microsoft excel 10 (*RANDBETWEEN* function). Numbers such as 60 or below will be assigned to diabetes conversation map (acronym DCM; *n* = 60) and numbers above 60 will be assigned to routine counseling (acronym RC; n = 60). Random numbers and their corresponding assignment will be sealed in the envelops, kept in sequence and open consecutively as patients are screened and found eligible for enrollment into the study. Principal Investigator (Rubina Qasim) of this project will open the envelopes for each participant to allocate the intervention.

### Study setting

This study will be conducted at National Institute of Diabetic and Endocrinology (NIDE) Ojha campus, located at Dow University of Health Sciences (DUHS). This is a public sector specialized teaching hospital with both inpatient and outpatient facilities. Average daily turnover of diabetic patients in the outpatient department is about more than 200. For those who require hospitalization, 250 bedded inpatient facilities are available. All the patients are assigned a unique medical record number and both laboratory, and essential medical records are electronically archived using this unique number. A subsidized fee for consultation and laboratory investigation is charged from affording patients. Welfare funds are used to pay the treatment cost of those who cannot afford to pay themselves.

### Study duration

This study will be completed in 6 months duration starting from November 25, 2018.

### Study population

#### Inclusion criteria

Patients aged 30–60 years, visiting the diabetes clinics of NIDE, diagnosed with T2DM for at least 5 years, HbA1c levels of > 7%, and found to be positive for diabetes distress (DD) based on validated DD screening tool (section 3 of Additional file 1) will be included in the study.

#### Exclusion criteria

Patients with T2DM with major disabilities, diagnosed with mental health problems/ cognitive pattern not intact, with major diabetes related complications, and living outside Karachi will be excluded from the study.

### Operational definitions

Diabetes control: Patients with T2DM with glycated hemoglobin (HbA1c) ≤7 will be considered as diabetes control.

Suboptimal control of diabetes: Patients with T2DM with HbA1c more than 7% at the time of enrollment will be considered suboptimal control of diabetes.

### Sample size

A study from China [17] reported mean DD score of control and intervention group at baseline as 32.77 ± 14.57 and 26.08 ± 9.92 respectively (*p* value 0.073). After six months of the intervention the respective scores were 30.09 ± 12.14 and 22.79 ± 4.95 (p value 0.014). Aiming for a higher average difference of the differences (difference of 7.30) as compared to the study from china (0.62) between the intervention and control arm (which is also clinically significant), considering 95% confidence interval and 80% power of the test to detect the given difference, the minimum sample size to achieve the objective of this study will be 88 (44 in each group). After adding attrition rate, a total of 120 subjects i.e. 60 in RC arm and 60 in DCM arm will be enrolled in this study.

### Enrollment and data collection procedure

The principle investigator (PI) will take permission from related authority. The PI will be sitting in the waiting area outside the clinic where the physician will refer all the patients diagnosed with T2DM with suboptimal management. After screening based on eligibility criteria, informed consent will be taken, and participant will be randomized. Structured Questionnaire translated in Urdu language and back translated in English will be used for collecting demographic, clinical, and laboratory data. After 3 months of enrollment, a follow up interview will be conducted using the same questionnaire and blood test for HbA1c to see the change in baseline measurements across the two groups.

#### Dependent variables

Diabetes Management Self-Efficacy (DMSE) and Diabetes Distress (DD) and HbA1c are dependent variable in this study.

#### Independent variables

This will include age, gender, BMI, education level, occupation, marital status, time since diabetes diagnosis, time since diabetes treatment, type of treatment for Diabetes e.g. Tablets, insulin etc. use of smart phones, use of internet, monthly income and intervention (DCM or RC).

#### Measurements of DMSE and DD

- The DMSE will be measured using validated DMSE scale (See Section 2 of Additional file 1) [18]. The scale has 20 items comprised of 4 domains; 1) nutrition specific and weight, 2) medical treatment, 3) physical exercise, 4) blood sugar. Each item is scored on 11-point Likert scale (0 = completely unable to 10 = completely able). Possible score ranges from 0 to 200, with higher score representing higher DMSE. The assessment of DMSE will be measured by taking mean scores and then compared at pre and post intervention.
- DD will be screened using validated DD scale (See Section 3 of Additional file 1) [19]. The scale has two parts; part 1 is consisting of two items asking about feelings of overburden due to demands of living with diabetes and feelings of failure with diabetes routine; the aim of part 1 is to screen for the presence of DD. Part 1 will be administered before the enrollment of the patient to screen for the presence of DD. If DD is present, part 2 will begin consisting of 17 items to score the extent of DD. Each item is scored on a Likert scale ranging from 1 (not a problem) to 6 (a very serious problem). According to the instructions of DD scoring sheet, total DD will be measured with mean score while dividing the sum of all items by 17. Similarly, mean scores of emotional burden (items 1, 3, 8, 11, 14), physician-related distress (items 2, 4, 9, 15), regimen-related distress (items 5, 6, 10, 12, 16) and interpersonal distress (items 7, 13, 17) will be measured by dividing the sums of respective items by respective numbers of items. The mean score of ≥3 will be the threshold for being distressed.

#### Measurement of HbA1c

Diabetic patients visiting NIDE are routinely advised for HbA1c to assess their diabetes control. These tests are performed at subsidized cost at the clinical laboratory of DUHS using the standardized methods. We will access the results of latest HbA1c tests at the time of screening and then 3 months after the enrollment into the study. HbA1c values will be categorized into two groups; 1) subjects where HbA1c < = 7 will be considered as patients with good diabetic control and 2) subjects where HbA1c > 7 will be considered as patients with sub-optimal diabetic control, therefore eligible for inclusion in the study.

#### Intervention and follow-ups

The intervention arm will receive 4 sessions based on DCM. These sessions will be conducted 1 week apart (45-60 min). These sessions will be facilitated by a trained educator. These sessions will include topics on managing diabetes, following a healthy lifestyle, starting insulin, and experiencing life with diabetes (Table 1).

**Table 1:** Diabetic education using diabetes conversation maps

Session − 1	How Diabetes Works
DCM − 1	This is a standard 3 by 5 ft colorful drawing which is used to teach patients with diabetes, how it occurs, and how to manage potential complications.
Duration	45–60 min
Content	Drawings of situations familiar to people with diabetes. The facilitator will use these drawings to help the patients sitting in group to understand the disease.
Session-2	Living with Diabetes
DCM − 2	This is a standard 3 by 5 ft colorful drawings used to teach patients about daily self-care, hypo or hyperglycemia, and psychosocial adjustment
Duration	45–60 min
Content	Drawings of different situations familiar to the diabetic patients.
Session-3	Healthy Eating and Keeping Active
DCM − 3	This is a standard 3 by 5 ft colorful drawings used to teach patients about healthy eating habits and lifestyle habits
Duration	45–60 min
Content	Provides information about dietary choices and portion sizes, along with different types of physical activity.
Session-4	Starting Insulin or oral antidiabetic medication
DCM − 4	This is a standard 3 by 5 ft colorful drawings used to teach patients about the use of Insulin or other oral medications.
Duration	45–60 min
Content	Focuses on the natural progression of diabetes, as well as insulin therapy and its potential benefits for people with type 2 diabetes.

In control group, participants will attend routine diabetes counseling provided by diabetic clinics’ trained nurses. These counseling are routinely provided in groups and can range from 30 to 60 min at the time of outpatient visit to a diabetic clinic. For comparability between the intervention and control arms, we will arrange four routine counseling sessions (one week apart). Detail flow of the study including follow-ups and measurements at different time-points is shown in Table 2.

**Table 2:**
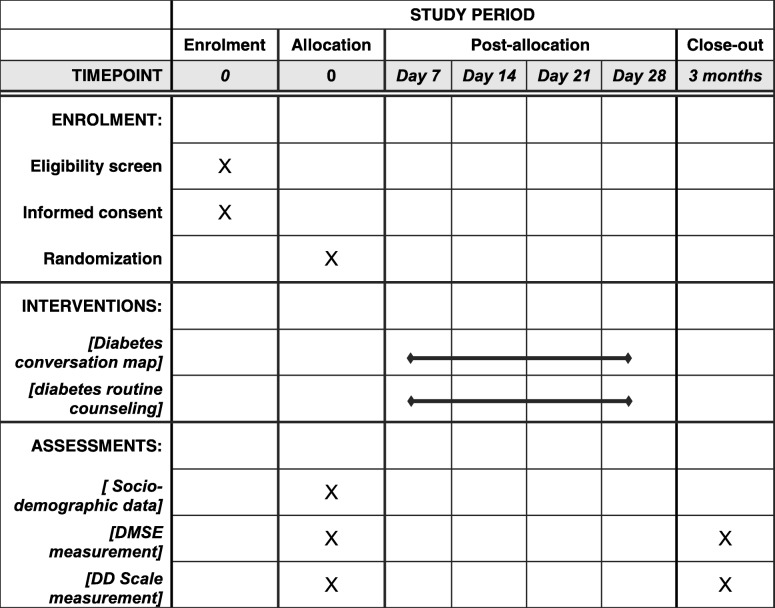
Checklist for the schedule of enrolment, interventions, and assessments

*DMSE* Diabetes management self-efficacy, *DD* Diabetes distress

### Ethical approval and consent to participate

This study is approved by the institutional review board (IRB) of the DDUHS, Karachi, Pakistan. Written informed consent will be obtained by the PI from all the participants. Data confidentiality will be maintained by keeping all questionnaires locked in a cabinet and electronic data will be kept in a password protected computer. Only the research team will have access to data.

### Plan of analysis

All statistical analysis will be performed using SPSS version 20. Intention to treat and per protocol analysis will be performed. Descriptive statistics will be reported by computing frequencies and percentages for categorical variables and means, standard deviation and ranges (minimum and maximum) values for continuous variables. All independent variables such as demographic and clinical characteristics will be compared at baseline between the two groups. Outcome variables will be calculated by summing the scores of respective scales. Paired sample t test will be used to measure the significant difference in HbA1C, DMSE and DD scores on a pre-post analysis in each group, followed by between the groups comparison using independent sample t test after the intervention. If the data collected through DMSE, DD and HbA1C will not follow the assumption of linearity for parametric testing (paired and independent sample T tests), we will use the non-parametric counterpart such as Wilcoxon signed-rank test and Mann-Whitney test, respectively. Similarly, if we could not enroll sufficient number of the participants or if there were few cases left for the analysis due to higher number of attrition rate, and the assumption for parametric testing is violated, we will switch to the non-parametric testing. If any characteristic such as demographic or clinical was found to be significantly different between the two groups at baseline, we will adjust the analysis for that variable using multivariable linear regression. Sensitivity analysis will be run to measure effect of any missing observations on outcome of the study.

## Discussion

The DCM education tools focus on four different domains related to successful diabetes self-management such as 1) living with diabetes, 2) how diabetes works, 3) healthy eating and keeping active, and 4) starting insulin. DCM based education guides people with diabetes through a process with the aim of helping them understand and internalize information about their disease and generate insightful conclusions, which may then result in improved self-management decisions and actions.

The initial draft of the DCM were tested in UK on a group of 56 patients with T2DM. Out of 56, 70% rated the sessions based on DCM as very effective helping them find something that would help them in controlling their diabetes, almost all the participants reported that they would recommend the learning experience with someone diagnosed with diabetes, and 98% wanted to learn more about the Conversation Map tools [20]. A pilot phased randomized controlled trial in China evaluated the effect of education based on DCM as compared to traditional education on DD and diabetes empowerment among patients with T2DM. The study reported significant reduction in DD score and significant increase in the diabetes empowerment score at six months after enrollment into the study among the patients who received education based on DCM as compared to the control arm. The study could not find any significant change in the HbA1C level at six months of enrollment between the intervention and control group [17]. In Pakistan, a cross sectional study conducted in a tertiary care hospital of Karachi showed that the use of DCM for teaching patients with T2DM was useful in improving the knowledge, attitude and practices of the patients [21]. Besides, no other study has been conducted in Pakistan or their neighboring countries to evaluate the effectiveness of these novel tools for DMSE and DD in well-designed, sufficiently powered clinical trials.

The major limitations of this study are that it will be conducted in a single public sector tertiary care hospital of Karachi and hence generalizability might be limited to the catchment population who visit public sector hospitals for their treatment. On the other hand, important strength of this study is the use of validated tools translated in local language for the assessment of DMSE and DD. In addition, this study will also measure change in the HbA1c level at baseline and 3 months after the enrollment to further confirm the benefit of the intervention in controlling diabetes.

## Additional file

Additional file 1: Questionnaire. (DOCX 29 kb)

## Data Availability

Can be available once collected.

## Abbreviations

DCM: Diabetes Conversation Map
DD: Diabetes distress
DMSE: Diabetes management self-efficacy
DUHS: Dow University of Health Sciences
HbA1c: Glycated hemoglobin (hemoglobin A1c)
IRB: Institutional Review Board
NIDE: National Institute of Diabetes and Endocrinology
SPSS: Statistical Package for Social Scientists
T2DM: Type 2 Diabetes Mellitus

## References

[1] NCD-RisC (2016). Worldwide trends in diabetes since 1980: a pooled analysis of 751 population-based studies with 4.4 million participants. Lancet.

[2] Whiting DR, Guariguata L, Weil C, Shaw J (2011). IDF diabetes atlas: global estimates of the prevalence of diabetes for 2011 and 2030. Diabetes Res Clin Pract.

[3] World Health Organization (2016). Global report on diabetes 2016.

[4] Funnell MM, Brown TL, Childs BP, Haas LB, Hosey GM, Jensen B (2009). National standards for diabetes self-management education. Diabetes Care.

[5] Williams BW, Kessler HA, Williams MV (2014). Relationship among practice change, motivation, and self-efficacy. J Contin Educ Heal Prof.

[6] Bandura A (1977). Self-efficacy: toward a unifying theory of behavioral change. Psychol Rev.

[7] Didarloo AR, Shojaeizadeh D, Gharaaghaji AR, Habibzadeh H, Niknami S, Pourali R (2012). Prediction of self-management behavior among Iranian women with type 2 diabetes: application of the theory of reasoned action along with self-efficacy (ETRA). Iran Red Crescent Med J.

[8] Polonsky WH, Anderson BJ, Lohrer PA (1995). Assessment of diabetes-related distress. Diabetes Care.

[9] Stoop CH, Nefs G, Pop VJ (2014). Diabetes-specific emotional distress in people with type 2 diabetes: a comparison between primary and secondary care. Diabet Med.

[10] Speight J, Browne JL, Holmes-Truscott E, Hendrieckx C, Pouwer F. Diabetes MILES – Australia 2011 survey report. Vic-Melbourne; 2011.

[11] Fisher L, Skaff MM, Mullan JT, Arean P, Glasgow R, Masharani U (2008). A longitudinal study of affective and anxiety disorders, depressive affect and diabetes distress in adults with type 2 diabetes. Diabet Med.

[12] Zagarins SE, Allen NA, Garb JL, Welch G (2012). Improvement in glycemic control following a diabetes education intervention is associated with change in diabetes distress but not change in depressive symptoms. J Behav Med.

[13] Fonda SJ, McMahon GT, Gomes HE, Hickson S, Conlin PR (2009). Changes in diabetes distress related to participation in an internet-based diabetes care management program and glycemic control. J Diabetes Sci Technol.

[14] Tan AS, Yong LS, Wan S, Wong ML (1997). Patient education in the management of diabetes mellitus. Singap Med J.

[15] Healthy Interactions. Creating the foundation for personal health engagement and self-management education. [cited January 08, 2017]. Available from: http://healthyinteractions.com/international-diabetes-conversation-map-education-tools-celebrates-one-year-anniversary.

[16] National Institute of Population Studies (NIPS) [Pakistan] and ICF. 2019. Pakistan Demographic and Health Survey 2017-18. Islamabad, Pakistan, and Rockville, Maryland, USA: NIPS and ICF.

[17] Li F, Yao P, Hsue C, Xu J, Lou Q (2016). Impact of “conversation maps” on diabetes distress and self-efficacy of Chinese adult patients with type 2 diabetes: a pilot study. Patient Preference Adherence.

[18] Sturt J, Hearnshaw H, Wakelin M (2010). Validation and reliability of the DMSE UK: a measure of self-efficacy for type 2 diabetes self-management. Primary Health Care Res Dev.

[19] Polonsky WH, Fisher L, Earles J, Dudl RJ, Lees J, Mullan J (2005). Assessing psychosocial distress in diabetes: development of the diabetes distress scale. Diabetes Care.

[20] Cradock S, Allard S, Moutter S (2010). Using conversation maps in practice: the UK experience. J Diabetes Nurs.

[21] Ghafoor E, Riaz M, Eichorst B, Fawwad A, Basit A (2015). Evaluation of diabetes conversation map™ education tools for diabetes self-management education. Diabetes Spectr.

