# Supplementary material for: Effect of diabetic counseling based on conversation map as compared to routine counseling on diabetes management self-efficacy and diabetic distress among patients with diabetes in Pakistan: a randomized controlled trial (study protocol)
Source: BMC Public Health. 2019 Jul 8;19:907. doi: 10.1186/s12889-019-7266-3 (PMC6615107; doi:10.1186/s12889-019-7266-3)
Supplement: Supplementary file 1 — Questionnaire. (DOCX 29 kb) [file 12889_2019_7266_MOESM1_ESM.docx]

**Additional file 1: Questionnaire**

**Section 1: Socio demographic characteristics**

|  | Participant MR # | ___________________ | | |
| --- | --- | --- | --- | --- |
|  | Subject ID | ___________________ | | |
|  | Name: |  | | |
|  | Date of filling the form(DD/MM/YY) |  | | |
|  | Date of Randomization(DD/MM/YY) |  | | |
|  | Group assigned | 1-----Group-A  2-----Group-B | | |
|  | Gender | 1-----Male  2-----Female | | |
|  | Age  (write Complete years) | ___________ years | | |
|  | Marital status | 1----Unmarried  2----Married  3----Divorced/Widow  4----Separated | | |
|  | Education | 1---No formal education  2---Primary  3---Secondary  4---Intermediate  5---Graduation  6---Master & above  7--- Others | | |
|  | Occupation  (write occupation) | ___________________________ | | |
|  | Household income | ________________________PKR | | |
|  | Time since DM diagnosis | _______ Years _________Months | | |
|  | Family history of DM | 1---Yes  2---No | | |
|  | Current Treatment | 1---Insulin  2---Oral Medicine  3---Others | | |
|  | Smoking | 1---Yes (currently smoking)  2---Yes (but quit earlier)  3---No | | |
|  | Tobacco use | 1---Yes (currently using)  2---Yes (but quit earlier)  3---No | | |
|  | Betal Nuts | 1---Yes (currently using)  2---Yes (but quit earlier)  3---No | | |
|  | Weight | _________kgs | | |
|  | Height | ________cm | | |
|  | Latest HBA1c | _________% | | |
|  | Level of Physical Activity | 1---Active  2---Sedentary | | |
|  | Other co-morbid | **Diseases** | **Yes** | **No** |
|  |  | 1---HTN | 1 | 2 |
|  |  | 2---Heb-B | 1 | 2 |
|  |  | 3---Hep-C | 1 | 2 |
|  |  | 4---Anemia | 1 | 2 |
|  |  | 5---renal Failure | 1 | 2 |
|  |  | 6---any Heart Disease | 1 | 2 |
|  |  | 7---Stroke | 1 | 2 |
|  |  | 8---others__________ | 1 | 2 |
|  | Diabetes Complications | 1---Neuropathy | 1 | 2 |
|  |  | 2---Retinopathy | 1 | 2 |
|  |  | 3--Nephropathy | 1 | 2 |
|  |  | 4---Vascularopathy | 1 | 2 |
|  |  | 5--Diabetic Foot | 1 | 2 |

**Section 2: Diabetes management self-efficacy scale (DMSE)**

*The following scale measure the self-efficacy related to different aspects of diabetes management among patients with diabetes.*

*Please read the following 20 statements and provide your response on a scale of 0 to 10. If you consider that certainly you cannot perform the particular task then you should circle “0”. If you are not certain whether you would be able to perform or not able to perform the task, you should circle “5”. For a task which you can certainly perform with full confidence, you should circle “10”.*

| # | Items description | Cannot do at all | | | | | May be yes/may be no | Certain can do | | | | |
| --- | --- | --- | --- | --- | --- | --- | --- | --- | --- | --- | --- | --- |
|  |  | 0 | 1 | 2 | 3 | 4 | 5 | 6 | 7 | 8 | 9 | 10 |
| 1 | I am able to check my blood/urine sugar if necessary |  |  |  |  |  |  |  |  |  |  |  |
| 2 | I am able to correct my blood sugar when the sugar level is too high |  |  |  |  |  |  |  |  |  |  |  |
| 3 | I am able to correct my blood sugar when the blood sugar level is too low |  |  |  |  |  |  |  |  |  |  |  |
| 4 | I am able to choose the correct food |  |  |  |  |  |  |  |  |  |  |  |
| 5 | I am able to choose different foods and stick to a healthy eating pattern |  |  |  |  |  |  |  |  |  |  |  |
| 6 | I am able to keep my weight under control |  |  |  |  |  |  |  |  |  |  |  |
| 7 | I am able to examine my feet for cuts |  |  |  |  |  |  |  |  |  |  |  |
| 8 | I am able to take enough exercise, for example, walking or riding a bicycle |  |  |  |  |  |  |  |  |  |  |  |
| 9 | I am able to adjust my eating plan when ill |  |  |  |  |  |  |  |  |  |  |  |
| 10 | I am able to follow a healthy eating pattern most of the time |  |  |  |  |  |  |  |  |  |  |  |
| 11 | I am able to take more exercise if the doctor advises me to |  |  |  |  |  |  |  |  |  |  |  |
| 12 | When taking more exercise I am able to adjust my eating plan |  |  |  |  |  |  |  |  |  |  |  |
| 13 | I am able to follow a healthy eating pattern when I am away from home |  |  |  |  |  |  |  |  |  |  |  |
| 14 | I am able to adjust my eating plan when I am away from home |  |  |  |  |  |  |  |  |  |  |  |
| 15 | I am able to follow a healthy eating pattern when I am on holiday |  |  |  |  |  |  |  |  |  |  |  |
| 16 | I am able to follow a healthy eating pattern when I am eating out or at a party |  |  |  |  |  |  |  |  |  |  |  |
| 17 | I am able to adjust my eating plan when I am feeling stressed or anxious |  |  |  |  |  |  |  |  |  |  |  |
| 18 | 18 I am able to visit my doctor once a year to monitor my diabetes |  |  |  |  |  |  |  |  |  |  |  |
| 19 | I am able to take my medication as prescribed |  |  |  |  |  |  |  |  |  |  |  |
| 20 | I am able to adjust my medication when I am ill |  |  |  |  |  |  |  |  |  |  |  |

**Section 3: Diabetes distress scale**

*Following are the two potential problem areas that people with diabetes may experience. Consider the degree to which each of the two items may have distressed you during the past six months and circle the appropriate number. If you feel that a particular item is not a problem for you, circle “1”. If it is very bothersome to you might opt to circle “6”.*

|  |  | Not a problem | A slight problem | A moderate problem | Somewhat serious problem | A serious problem | A very serious problem |
| --- | --- | --- | --- | --- | --- | --- | --- |
| 1 | Feeling overwhelmed by the demand of living with diabetes | 1 | 2 | 3 | 4 | 5 | 6 |
| 2 | Feeling that I am often failing with my diabetes routine | 1 | 2 | 3 | 4 | 5 | 6 |

*If the above two items are problematic for you and your responses are not “1”, then please start answering the following 17 questions as well. In case the responses to the above two items are “1” then you can skip the following 17 questions.*

|  |  | Not a problem | A slight problem | A moderate problem | Somewhat serious problem | A serious problem | A very serious problem |
| --- | --- | --- | --- | --- | --- | --- | --- |
| 1 | Feeling that diabetes is taking up too much of my mental and physical energy every day | 1 | 2 | 3 | 4 | 5 | 6 |
| 2 | Feeling that my doctor doesn’t know enough about my diabetes care | 1 | 2 | 3 | 4 | 5 | 6 |
| 3 | Feeling angry, scared and or depressed when I think about living with diabetes. | 1 | 2 | 3 | 4 | 5 | 6 |
| 4 | Feeling that my doctor doesn’t give me clear enough direction on how to manage my diabetes. | 1 | 2 | 3 | 4 | 5 | 6 |
| 5 | Feeling that I am not testing my blood sugar frequently enough | 1 | 2 | 3 | 4 | 5 | 6 |
| 6 | Feeling that I am often failing with my diabetes routine. | 1 | 2 | 3 | 4 | 5 | 6 |
| 7 | Feeling that friends or family are not supportive enough of self-care efforts (e.g. planning activities that conflict with my schedule, encouraging me to eat the “wrong” foods) | 1 | 2 | 3 | 4 | 5 | 6 |
| 8 | Feelings that diabetes controls my life | 1 | 2 | 3 | 4 | 5 | 6 |
| 9 | Feeling that my doctor doesn’t take my concerns seriously enough. | 1 | 2 | 3 | 4 | 5 | 6 |
| 10 | Not feeling confident in my day-to-day ability to manage diabetes. | 1 | 2 | 3 | 4 | 5 | 6 |
| 11 | Feeling that I will end up with serious long-term complications, no matter what I do. | 1 | 2 | 3 | 4 | 5 | 6 |
| 12 | Feeling that I am not sticking closely enough to a good meal plan. | 1 | 2 | 3 | 4 | 5 | 6 |
| 13 | Feeling that friends or family don’t appreciate how difficult living with diabetes can be | 1 | 2 | 3 | 4 | 5 | 6 |
| 14 | Feeling overwhelmed by the demands of living with diabetes | 1 | 2 | 3 | 4 | 5 | 6 |
| 15 | Feelings that I don’t have a doctor who I can see regularly enough about my diabetes. | 1 | 2 | 3 | 4 | 5 | 6 |
| 16 | Not feeling motivated to keep up my diabetes self-management. | 1 | 2 | 3 | 4 | 5 | 6 |
| 17 | Feeling that friends or family donot give me the emotional support that I would like. | 1 | 2 | 3 | 4 | 5 | 6 |
